# Supplementary material for: Molecular basis for variations in the sensitivity of pathogenic rhodopsin variants to 9-cis-retinal
Source: J Biol Chem. 2022 Jul 16;298(8):102266. doi: 10.1016/j.jbc.2022.102266 (PMC9399271; doi:10.1016/j.jbc.2022.102266)
Supplement: Supplementary Information [file mmc1.pdf]

Supplementary Information for:

Molecular Basis for Variations in the Sensitivity of Pathogenic Rhodopsin Variants to 9-*cis*-Retinal

Francis J. Roushar,[1†] Andrew G. McKee,[1†] Charles P. Kuntz,[1] Joseph T. Ortega,[2] Wesley D. Penn,[1] Hope Woods,[3,4] Laura M. Chamness,[1] Victoria Most,[5] Jens Meiler,[3,5] Beata Jastrzebska,[2] and Jonathan P. Schlebach[1]*

[1]*Department of Chemistry, Indiana University, Bloomington, Indiana, USA*
[2]*Department of Pharmacology, Case Western Reserve University, Cleveland, OH, USA*
[3]*Department of Chemistry, Vanderbilt University, Nashville, TN, USA*
[4]*Chemical and Physical Biology Program, Vanderbilt University, Nashville, TN, USA*
[5]*Institute for Drug Development, Leipzig University, Leipzig, SAC, Germany*

Keywords: Membrane Protein Folding, Rhodopsin, Proteostasis, Corrector, GPCR

†Authors contributed equally
**Corresponding author* E-mail address: jschleba@indiana.edu

Contents:
-Figure S1
-Table S1

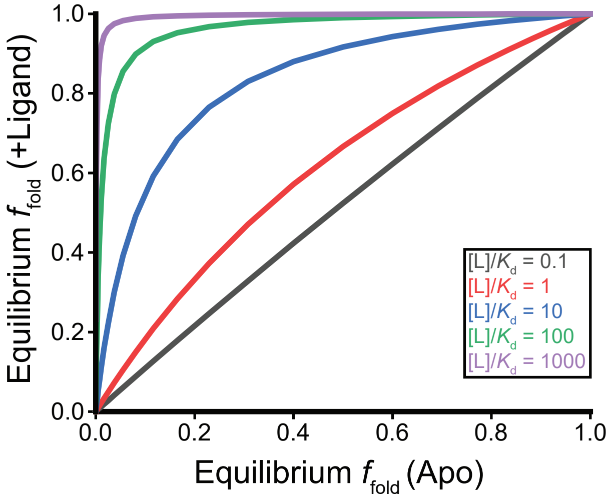

Figure S1. Thermodynamic Relationship Between Ligand Binding on the Equilibrium Fraction of Folded Protein. Equation 5 was used to similate the impact of ligand binding on the equilibrium fraction of folded protein as a function of the ratio of ligand to its equilibrium dissociation constant. The equilibrium fraction of folded protein in the presence of ligand is plotted as a function of corresponding equilibrium fraction of folded apoprotein. This simulation reveals how the degree of rescue offered by a corrector should relate to the thermodynamic stability of the variant (x-coordinate), the concentration of the ligand, and its binding affinity.

Table S1. Classification, Retinal-Sensitivity, and Plasma Membrane Expression (PME) of Mutations in Rhodopsin

Mutation	Prior Classification	DMS Classification	Surface Immunostaing Intensity (Apo)*	Surface Immunostaing Intensity (+Ret)*
T4K	Class 2[1, 2]	Non-Class 2	21459 ± 1373	19838 ± 3633
P12R	Unclassified	Moderate Class 2	3044 ± 30	5245 ± 834
N15S	Class 2[1]	Severe Class 2	2058 ± 88	2473 ± 303
T17M	Class 2[1-3]	Severe Class 2	2211 ± 75	2604 ± 227
V20G	Class 2[1]	Severe Class 2	2251 ± 91	2774 ± 268
P23H	Class 2[1-3]	Severe Class 2	1716 ± 7	2133 ± 171
P23L	Class 2[1]	Severe Class 2	1585 ± 55	1988 ± 255
P23A	Class 2[1]	Severe Class 2	2295 ± 24	2920 ± 275
Q28H	Class 2[1, 2]	Severe Class 2	1905 ± 82	2236 ± 102
Q28R	Class 2[1]	Severe Class 2	1826 ± 51	2203 ± 117
M39R	Class 4[1]	Moderate Class 2	7406 ± 61	8483 ± 646
M44T	Class 5[1]	Non-Class 2	20520 ± 250	22775 ± 1128
F45L	Class 7[1]	Non-Class 2	21466 ± 261	23900 ± 1331
L46R	Unclassified	Severe Class 2	1575 ± 55	1887 ± 86
L47R	Class 2[4]	Moderate Class 2	2891 ± 92	5580 ± 159
G51R	Class 2[1, 2, 4]	Moderate Class 2	6840 ± 236	11251 ± 1695
G51A	Unclassified	Non-Class 2	19022 ± 278	21907 ± 945
G51V	Class 2[1, 2]	Non-Class 2	19866 ± 375	21197 ± 624
F52Y	Unclassified	Moderate Class 2	12693 ± 203	16135 ± 2076
P53R	Class 2[1, 2]	Severe Class 2	1659 ± 75	3510 ± 247
F56Y	Unclassified	Non-Class 2	16591 ± 109	18485 ± 1691
L57R	Class 2[4]	Moderate Class 2	2956 ± 128	4650 ± 184
T58R	Class 2[1, 3, 4]	Non-Class 2	17889 ± 209	22138 ± 1739
R69H	Unclassified	Non-Class 2	20184 ± 589	23143 ± 999
del N73	Unclassified	Moderate Class 2	4913 ± 293	11881 ± 2355
N78I	Class 2[4]	Moderate Class 2	3185 ± 274	4998 ± 625
V87L	Unclassified	Non-Class 2	18186 ± 779	22577 ± 1112
V87D	Class 2[1-3]	Moderate Class 2	3731 ± 472	5216 ± 642
L88P	Class 2[4]	Moderate Class 2	7799 ± 124	12400 ± 2582
G89D	Class 2[1-4]	Moderate Class 2	10580 ± 514	9423 ± 1590
G90D	Class 6[1]	Non-Class 2	21297 ± 242	20572 ± 503
G90V	Class 6[1]	Non-Class 2	21101 ± 268	20872 ± 1098
T94I	Class 6[1]	Non-Class 2	25614 ± 185	24016 ± 497
V104I	Unclassified	Non-Class 2	20746 ± 333	23099 ± 1134
V104F	Unclassified	Non-Class 2	17108 ± 351	18678 ± 550
G106R	Class 2[1, 2]	Moderate Class 2	6597 ± 210	8280 ± 1114
G106W	Class 2[1-3]	Severe Class 2	2101 ± 99	2993 ± 315
G109R	Class 2[4]	Moderate Class 2	14723 ± 150	13588 ± 2744
C110R	Class 2[1]	Severe Class 2	1645 ± 73	2062 ± 114
C110Y	Class 2[1, 2]	Severe Class 2	1660 ± 86	2036 ± 96
C110F	Class 2[1]	Severe Class 2	1571 ± 80	1920 ± 127
G114D	Unclassified	Severe Class 2	1663 ± 103	1958 ± 81
G114V	Unclassified	Severe Class 2	1575 ± 41	2544 ± 155
E122G	Unclassified	Non-Class 2	21147 ± 314	22740 ± 1211

Mutation	Prior Classification	DMS Classification	Surface Immunostaing Intensity (Apo)*	Surface Immunostaing Intensity (+Ret)*
L125R	Class 2[1]	Non-Class 2	19889 ± 220	18451 ± 1729
S127F	Unclassified	Moderate Class 2	2693 ± 178	3591 ± 326
L131P	Unclassified	Severe Class 2	1882 ± 130	3211 ± 325
R135P	Class 3[1]	Moderate Class 2	8559 ± 202	13039 ± 2177
R135L	Class 2[3]	Moderate Class 2	4085 ± 130	7931 ± 378
R135G	Class 3[1]	Moderate Class 2	8791 ± 181	14820 ± 2169
R135W	Class 2[3]	Severe Class 2	2129 ± 65	7701 ± 299
V137M	Class 5[1]	Non-Class 2	20724 ± 216	22958 ± 621
C140S	Unclassified	Non-Class 2	20898 ± 309	23562 ± 1372
E150K	Unclassified	Non-Class 2	20855 ± 324	23140 ± 993
T160T	Unclassified	Non-Class 2	20350 ± 359	22847 ± 1185
W161R	Class 2[1]	Severe Class 2	1812 ± 54	2330 ± 231
A164E	Class 2[1]	Severe Class 2	1603 ± 106	2606 ± 219
A164V	Class 2[1]	Non-Class 2	18661 ± 308	20327 ± 713
C167R	Class 2[1, 2, 4]	Non-Class 2	20651 ± 320	23214 ± 1035
C167W	Class 2[1, 4]	Severe Class 2	1904 ± 28	3093 ± 197
A169P	Class 2[4]	Severe Class 2	1475 ± 67	2386 ± 119
P170R	Unclassified	Severe Class 2	1461 ± 10	1926 ± 283
P171E	Unclassified	Severe Class 2	1738 ± 91	2465 ± 117
P171Q	Class 2[1]	Severe Class 2	1675 ± 74	2429 ± 123
P171L	Class 2[1, 2]	Severe Class 2	1874 ± 87	2679 ± 177
G174S	Unclassified	Non-Class 2	19427 ± 265	23140 ± 82
S176F	Unclassified	Severe Class 2	1649 ± 36	2045 ± 212
Y178N	Class 2[1]	Severe Class 2	1622 ± 73	2916 ± 178
Y178D	Class 2[1]	Severe Class 2	1604 ± 83	2142 ± 38
I179F	Class 2[4]	Moderate Class 2	4667 ± 55	7646 ± 964
P180A	Class 2[4]	Moderate Class 2	3883 ± 159	6555 ± 480
P180S	Class 2[4]	Severe Class 2	1667 ± 76	2127 ± 123
E181K	Class 2[1, 2]	Severe Class 2	1705 ± 42	2236 ± 25
G182S	Class 2[1]	Moderate Class 2	3154 ± 213	5613 ± 471
Q184P	Unclassified	Severe Class 2	1852 ± 41	2236 ± 126
C185R	Class 2[1]	Severe Class 2	1816 ± 28	2271 ± 319
S186P	Class 2[2]	Severe Class 2	1582 ± 51	1991 ± 137
S186W	Class 2[4]	Non-Class 2	27766 ± 317	28230 ± 875
C187Y	Class 2[1]	Severe Class 2	1876 ± 64	2166 ± 54
G188R	Class 2[1, 2]	Severe Class 2	1660 ± 66	2113 ± 289
D190N	Class 2[1]	Moderate Class 2	4182 ± 24	5586 ± 797
D190G	Class 2[1, 3]	Severe Class 2	1788 ± 76	2442 ± 115
D190Y	Class 2[1, 2]	Severe Class 2	1757 ± 98	2216 ± 130
Y191C	Unclassified	Non-Class 2	17171 ± 396	22547 ± 458
T193M	Class 2[4]	Moderate Class 2	12826 ± 604	17646 ± 1170
P196T	Unclassified	Non-Class 2	19201 ± 404	22146 ± 1183
I205S	Unclassified	Non-Class 2	16477 ± 104	19163 ± 538
M207K	Unclassified	Non-Class 2	18429 ± 352	17930 ± 1375
M207R	Class 2[4]	Non-Class 2	15790 ± 243	13919 ± 804

Mutation	Prior Classification	DMS Classification	Surface Immunostaing Intensity (Apo)*	Surface Immunostaing Intensity (+Ret)*
V209M	Class 7[1]	Non-Class 2	18653 ± 98	21664 ± 690
V210F	Unclassified	Non-Class 2	19755 ± 141	22284 ± 776
H211R	Class 2[1]	Severe Class 2	1842 ± 5	2237 ± 181
I214N	Class 2[4]	Non-Class 2	17234 ± 484	19082 ± 1133
P215T	Unclassified	Severe Class 2	1521 ± 78	1972 ± 215
P215L	Unclassified	Severe Class 2	2307 ± 112	3037 ± 207
M216K	Class 2[4]	Non-Class 2	17663 ± 243	20170 ± 705
M216L	Unclassified	Non-Class 2	21093 ± 31	23273 ± 1180
F220C	Class 7[1]	Non-Class 2	19781 ± 348	22066 ± 605
R252P	Unclassified	Non-Class 2	18510 ± 536	20364 ± 182
del I255	Unclassified	Severe Class 2	1936 ± 36	2844 ± 225
del C264	Unclassified	Severe Class 2	1994 ± 106	2291 ± 53
G284S	Unclassified	Moderate Class 2	13474 ± 730	14941 ± 2034
T289P	Unclassified	Moderate Class 2	2357 ± 197	4688 ± 301
A295V	Class 6[1]	Non-Class 2	21819 ± 280	22891 ± 1079
S297R	Unclassified	Severe Class 2	1638 ± 41	2208 ± 185
A298D	Unclassified	Non-Class 2	17093 ± 306	20131 ± 2116
K311E	Unclassified	Non-Class 2	19549 ± 358	21928 ± 1173
T320N	Unclassified	Non-Class 2	20522 ± 116	22270 ± 1060
L328P	Class 1[1]	Non-Class 2	22042 ± 342	24896 ± 974
A333V	Unclassified	Moderate Class 2	12407 ± 392	14555 ± 433
E341K	Class 2[1]	Non-Class 2	16622 ± 95	17258 ± 2084
T342M	Class 1[1]	Non-Class 2	24656 ± 401	28184 ± 1242
S343C	Unclassified	Non-Class 2	19372 ± 323	21627 ± 993
Q344P	Class 1[1]	Non-Class 2	17471 ± 257	18834 ± 1694
Q344R	Class 1[1]	Non-Class 2	19609 ± 277	22090 ± 1221
V345M	Class 1[1]	Non-Class 2	21159 ± 313	23618 ± 1023
V345L	Class 1[1]	Non-Class 2	20578 ± 243	23057 ± 1079
A346P	Class 1[1]	Non-Class 2	20301 ± 335	22845 ± 1338
P347Q	Class 1[1]	Non-Class 2	20697 ± 270	23161 ± 1184
P347R	Class 1[1]	Non-Class 2	19849 ± 272	22682 ± 1304
P347L	Class 1[1]	Non-Class 2	22253 ± 148	24541 ± 869
P347A	Class 1[1]	Non-Class 2	18548 ± 679	21170 ± 753
P347S	Class 1[1]	Non-Class 2	20830 ± 291	23864 ± 986

[1] Athanasiou, D., Aguila, M., Bellingham, J., Li, W., McCulley, C., Reeves, P. J., and Cheetham, M. E. (2018) The molecular and cellular basis of rhodopsin retinitis pigmentosa reveals potential strategies for therapy, *Progress in Retinal and Eye Research 62*, 1-23.

[2] Kaushal, S., and Khorana, H. G. (1994) Structure and function in rhodopsin. 7. Point mutations associated with autosomal dominant retinitis pigmentosa, *Biochemistry 33*, 6121-6128.

[3] Sung, C. H., Schneider, B. G., Agarwal, N., Papermaster, D. S., and Nathans, J. (1991) Functional heterogeneity of mutant rhodopsins responsible for autosomal dominant retinitis pigmentosa, *Proceedings of the National Academy of Sciences 88*, 8840.

[4] Wan, A., Place, E., Pierce, E. A., and Comander, J. (2019) Characterizing variants of unknown significance in rhodopsin: A functional genomics approach, *Human Mutation 40*, 1127-1144.
